# Lubricant Strategies in Osteoarthritis Treatment: Transitioning from Natural Lubricants to Drug Delivery Particles with Lubricant Properties

**DOI:** 10.3390/jox14030072

**Published:** 2024-09-19

**Authors:** Agnese Fragassi, Antonietta Greco, Roberto Palomba

**Affiliations:** 1 Department of Pharmaceutical and Pharmacological Sciences, University of Padova, Via F. Marzolo 5, 35131 Padova, Italy; 2 Department of Medicine and Surgery, NanoMedicine Center (NANOMIB), University of Milano-Bicocca, Via Follereau 3, 20854 Vedano al Lambro, Italy; 3 Laboratory of Nanotechnology for Precision Medicine, Italian Institute of Technology, Via Morego 30, 16163 Genova, Italy

**Keywords:** osteoarthritis treatment, cartilage lubrication, biomimetic polymers, hydrogels

## Abstract

Osteoarthritis (OA) is a debilitating joint disease characterized by cartilage degradation, leading to pain and functional impairment. A key contributor to OA progression is the decline in cartilage lubrication. In physiological conditions, synovial fluid (SF) macromolecules like hyaluronic acid (HA), phospholipids, and lubricin play a crucial role in the boundary lubrication of articular cartilage. In early OA, cartilage damage triggers inflammation, altering SF composition and compromising the lubrication layer. This increases friction between mating interfaces, worsening cartilage degradation and local inflammation. Therefore, early-stage restoration of lubrication (by injecting in the joint different classes of compounds and formulations) could alleviate, and potentially reverse, OA progression. In the light of this, a broad variety of lubricants have been investigated for their ability to reduce friction in OA joints and promote cartilage repair in clinical and preclinical studies. This review examines recent advancements in lubricant-based therapy for OA, focusing on natural, bioinspired, and alternative products. Starting from the currently applied therapy, mainly based on natural lubricants as HA, we will present their modified versions, either in hydrogel form or with specific biomimetic moieties with the aim of reducing their clearance from the joint and of enhancing their lubricating properties. Finally, the most advanced and recent formulation, represented by alternative strategies, will be proposed. Particular emphasis will be placed on those ones involving new types of hydrogels, microparticles, nanoparticles, and liposomes, which are currently under investigation in preclinical studies. The potential application of particles and liposomes could foster the transition from natural lubricants to Drug Delivery Systems (DDSs) with lubricant features; transition which could provide more complete OA treatments, by simultaneously providing lubrication replacement and sustained release of different payloads and active agents directly at the joint level. Within each category, we will examine relevant preclinical studies, highlighting challenges and future prospects.

## 1. Introduction

Lubrication within synovial joints is essential for reducing friction between cartilage surfaces, whose preservation is crucial for ensuring smooth articular movements in everyday life [1]. A healthy articular cartilage is a self-lubricating system; the synovial fluid (SF) filling the joint cavity contains precise concentrations of several macromolecules: hyaluronic acid (HA), lubricin, and phospholipids (PL), which are all synergically involved in this process [2]. An altered lubrication of the synovial joint is considered one of the major contributors to the progressive degradation of articular cartilage found in osteoarthritis (OA), pathology which is widely considered the most common degenerative joint disease [3]. The triggering event of the disease is generally the initial articular cartilage damage, leading to the alteration of biolubricant composition [4]. Although the exact sequence of events causing cartilage degradation is not fully understood, it is widely recognized that the resulting increase in intra-articular (IA) friction plays a key role in worsening the pathology. Specifically, an increased friction exacerbates synovial inflammation which further contributes to cartilage degradation [5,6]. To interrupt this degenerative loop mechanism, restoring and maintaining adequate cartilage lubrication has emerged as a promising strategy in managing and potentially reversing OA progression [7]. With this purpose, various lubrication-based strategies have been tested, paving the way for innovative OA treatments [8,9]. Three main groups of therapeutics were generated and investigated: (i) natural biolubricants, primarily HA, (ii) bioinspired lubricants, and (iii) alternative lubricants. The two first derive from the application of exogenous molecules, either in their native form, or in modified ones (with the aim to improve their performances). The alternative lubricants instead include: hydrogels, microparticles (MP), nanoparticles (NP), and liposomes. Particles and liposomes can also serve as Drug Delivery Systems (DDSs) employing a dual-purpose approach aimed at tackling inflammation and friction at the same time [10]. This review provides an overview of joint physiology, followed by an in-depth examination of the most recent advancements in lubricant-based therapies for OA. A specific focus will be given to those DDSs able to grant at the same time the needed lubrication and the release of different payloads directly at the joint level.

## 2. Structural Components of Articular Joints

The primary structural and biological components of joints are cartilage, SF, and synovium. Articular cartilage, a highly specialized connective tissue found in diarthrodial joints (such as hips and knees) is a very thin layer. In humans, its thickness is only about 1–3 mm [11]. This pliable and elastic surface lacks blood and lymphatic vessels, and it is not innervated. Articular cartilage primary function is to offer a smooth and lubricated surface for the transmission of loads with minimal frictional coefficient [12]. Healthy cartilage is populated by chondrocytes, whose role is to produce and maintain the extracellular matrix (ECM) which, at the same time, represents the main constituent of the cartilage itself [13]. Excluding water, the main constituents of the ECM are collagen, proteoglycans (PGs), PL, and other macromolecules, all produced by chondrocytes. Collagen (mostly type II) accounts for approximately 60% of the dry weight of the cartilage and has primarily a structural role. Its fibers form a cross-linked core network which contributes to the mechanical stability of the matrix, endowing articular cartilage with significant shear and tensile properties [14]. Collagen networks interweave with PGs, the second most abundant macromolecules in articular cartilage. PGs (as, for example, aggrecan) are a family of glycoconjugates with a central core protein, to which one or more glycosaminoglycans (GAGs) (chondroitin sulfate and keratan sulfate) side chains are covalently linked [15]. GAGs are negatively charged because of the presence of the –SO_3_^−^ or –COO^−^ group. These negative charges retain large amounts of water molecules which furtherly strengthen cartilage matrix [16]. Besides collagen and PGs, another particularly important ECM constituent produced by chondrocytes is HA. This molecule is the only non-sulfated GAG (6500–10,900 kDa) and it is constituted by repeating disaccharide units of D-glucuronic acid and D-N-acetylglucosamine attached by β (1–4) and β (1–3) glycosidic bonds [17]. It forms non-covalent bounds with most of the PGs present in the matrix (as for example aggrecan), thus creating a PG backbone with 3 functions: (i) protecting the cartilage, (ii) blocking the loss of PGs from the cartilage matrix into the synovial space, and (iii) maintaining the physical form of the ECM [18].

Spatially, articular cartilage can be subdivided into three zones, characterized by a different composition: the superficial zone, the middle or transitional zone, and the deep zone [19]. The superficial zone (10–20% of the cartilage volume) is characterized by the presence of HA, mucine-like glycoprotein lubricin, and PL (all also present in the SF filling the joint cavity) [20]. In this area, the collagen content is the highest, and collagen fibers (predominantly type II) are aligned in parallel to the articular surface, enhancing resistance to compressive, tensile, and shear forces. The middle (or transitional zone) represents 40–60% of the cartilage volume. It contains obliquely organized collagen fibers and more spherical, sparsely distributed chondrocytes [21]. The deep zone constitutes the remaining ~30% of the articular cartilage volume. In this portion, the collagen is characterized by fibrils of large diameter perpendicularly oriented to the tidemark (the boundary between the uncalcified and the calcified cartilage adjacent to the subchondral bone) [22]. Due to the lack of blood vessel, oxygen and nutrients diffuse into the cartilage tissue directly from the SF, which is responsible for cartilage biolubrication and sustainment [23]. SF is lined by the synovium which, together with the subchondral bone, contributes to the nutrition of the articular cartilage. The synovium comprises two segments: an inner layer of cells (intima) and the underlying tissue (subintima) [18,24]. The intima contains two distinct cell types: type A macrophage-like synoviocytes (MLS) and type B fibroblast-like synoviocytes (FLS). MLS, derived from primitive macrophages present in the yolk sac, are phagocytic cells characterized by numerous lysosomes, removing debris from the joint space and serving as sentinels against microbial invasion [25]. FLS are mesenchymal cells with abundant rough endoplasmic reticulum, responsible for producing the ECM that supports the synovial lining. They also secrete hyaluronan and lubricin, essential components of SF, fundamental for joint lubrication [26]. The outer layer, known as the subintima, is comprised of three types of connective tissues: fibrous (dense collagenous type), adipose (predominantly found in fat pads), and areolar [23].

## 3. Articular Cartilage Lubrication Mechanism

**Figure 1:**
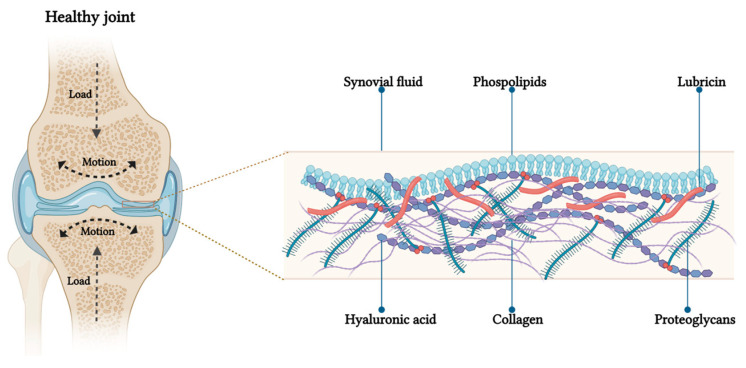
Schematic representation of boundary lubrication in articular cartilage. On the left, a coronal section of knee joint: under load application the pressure increases and the outer surfaces of opposing articular cartilage make molecular contact. On the right, the proposed structure of the lubrication layer on the cartilage surface, showing phosphocholine groups of lipids at the interface to reduce friction through the hydration mechanism.

In primates, the major weight-bearing joints, such as the hips and knees, possess a remarkable capability to support loads and function as high-efficiency tribological systems with low friction levels [27]. Friction, as a parameter, can be measured using the ‘coefficient of friction’ $\mu =\frac{f}{N}$, which represents the ratio between the force needed to slide the surfaces (f) and the load compressing those surfaces (N). In the context of the joint, μ can be measured for the two opposing cartilage surfaces found in the articular cavity. In vivo, joint surfaces experience modest sliding speeds during activities such as walking, ranging from rest (0 m/s) to approximately 0.3 m/s [28]. In terms of shear rates, daily movements, such as walking, generate local shear rates as high as 10^6−7^ m/s at the slip plane, where the sheared interfacial region is around 10 nm thick. In its healthy state, articular cartilage operates as a self-lubricating and self-healing system, preserving low friction coefficients between the opposing sliding surfaces of the joints. Friction coefficients (µ) for normal whole synovial joints have been reported in the range of μ ≈ 0.002–0.02 [29]. The capacity of having such a low µ is crucial to avoid cartilage erosion and its downstream effects. For decades, researchers have extensively investigated the mechanism through which the friction coefficient between healthy cartilages results extremely low. Friction and pressure distribution studies on the surface of the articular cartilage showed that joint lubrication involves a combined effect of both fluid film and boundary lubrications [30,31]. Fluid film lubrication predominates at low loads and high sliding speeds, while boundary lubrication dominates at high applied loads and low sliding speeds. In the first scenario, interstitial fluid is extruded to form a film between the surfaces. This pressurized fluid supports most of the normal load, reducing the mechanical stress on the cartilage matrix and resulting in low friction [32]. In the second scenario, under higher pressure, the fluid film is squeezed out, leading to the predominance of boundary lubrication. In boundary lubrication, friction arises from the partial contact between the two opposing cartilage surfaces. The friction coefficient is relatively unaffected by the sliding speed and normal load, and is primarily determined by the outer surface of the articular cartilage and the molecules within the SF [33]. The presence of biolubricants is particularly crucial in those situations in which cartilage results compressed under a severe joint loading. Among others, the most important biolubricants are: HA, GAG, lubricin, and PL [31,34]. Several investigations showed that the synergy between these molecules in the SF is more important than the contribution of the single one, and it is responsible for an efficient lubrication of articular cartilage under severe joint loading [34]. Specifically, the lubricin found in the outer superficial zone, or absorbed on the articular surface, interacts with HA. The so-complexed HA also binds PL monolayers and bilayers [35]. This interaction has been suggested to be hydrophobic (between the acyl chains in the PL and the hydrophobic patches in the HA polymer chain), hydrophilic (between the dipolar phosphocholine head groups and the negatively charged HA chain) or a combination of the two [36]. By actively interacting both with lubricin and PL, HA can be considered the physiological backbone of the SF biolubricant. This specific conformation of the three components synergistically creates low friction conditions inside the joints at their physiologically high pressures, via a hydration-lubrication mechanism (Figure 1). The hydration-lubrication mechanism is an energy dissipation process based on hydration. In this specific case, water molecules are tenaciously attached by the zwitterionic structure of the phosphocholine groups, without being squeezed out under large normal joint stress [35]. Despite this bound, water molecules remain fluid and can act as a shell for the two surfaces by sliding past each other or past surfaces [37]. The maintenance of this complex system, allowing both for cushioning and lubrication, strictly depends on articular cartilage homeostasis which is mainly regulated by the cartilage chondrocytes, and synovium synoviocytes and fibroblasts.

## 4. The Role of Cartilage Lubrication in Osteoarthritis Pathogenesis

**Figure 2:**
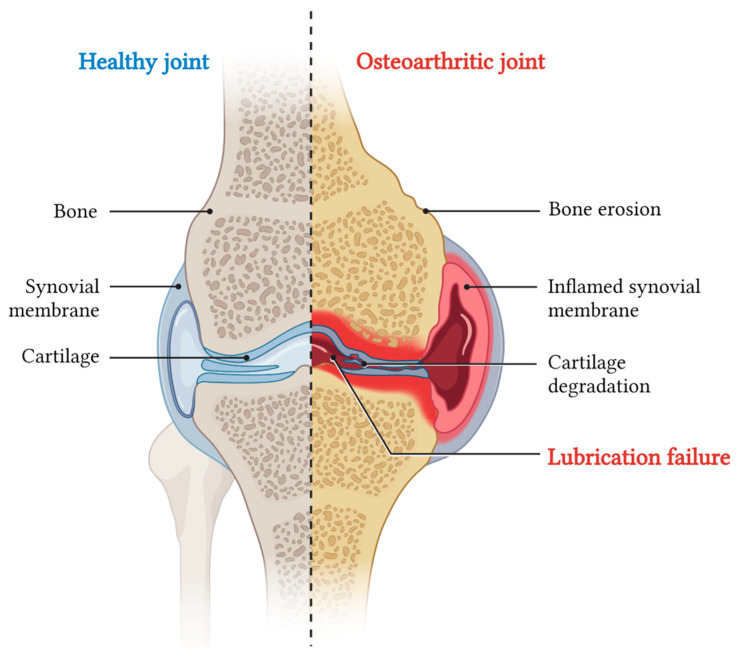
Schematic of a coronal section of knee joint indicating on the left the main anatomical components, and on the right the main pathological effects of OA.

When exposed to prolonged stress, chondrocytes fail to maintain cartilage homeostasis, thus increasing the risk of developing OA [4]. This pathology is currently considered the most prevalent joint disorder and, at the same time, the leading cause of pain and disability. The progressive cartilage degradation observed in the pathology is fostered by catabolic enzymes, and it is linked to the inflammation of the synovial membrane (synovitis), and other conditions which are difficult to revert, such as cartilage calcification, bone erosion, and subchondral bone sclerosis (Figure 2) [38]. 

Globally, over 595 million people are affected by OA, with higher prevalence among elderly individuals and women [39]. This high incidence imposes a substantial economic burden on national healthcare systems and society [40]. Nevertheless, treatment options for OA remain limited. Conventional therapy primarily involves IA injections of drugs, predominantly focused on alleviating the symptoms (pain, stiffness, swelling) while only temporary limiting cartilage degradation processes. Usually, these approaches also ameliorate the joint load tolerance by acting as a cushion, but the effect only lasts the time of retention of the injected formulations [41].

To date, no clinically available disease-modifying OA drugs, capable of reverting the cartilage damage, are available [42]. As mentioned above, OA pathogenesis is governed by multiple factors, and at present, the exact sequence of events that leads to cartilage degradation is not fully defined. However, it was clearly documented that OA disease is driven by an intricated conjunction of mechanical, cellular, and molecular factors that converge on the local production of pro-inflammatory cytokines and catabolic enzymes [43]. The three factors contribute to the gradual degradation of cartilage and, in absence of treatment, the additive effect one has on the others easily worsens the progressively insufficient cartilage lubrication. Indeed, studies conducted on various animal models of OA and in patients have shown the intimate correlation between the disruption of cartilage surface integrity and alterations in SF biolubricant composition. Kosinska et al. found that, compared to control SF, the concentrations of HA and lubricin were lower in SF from patients with OA and rheumatoid arthritis (RA), while PL concentrations were higher in these conditions (Table 1). Besides their composition, another interesting result was the finding that SF HA molecular weight (MW) also resulted as altered in OA patients. MW distribution of HA in OA and RA SF shifted toward lower ranges. The available information points toward recognizing that, among others, one of the major SF alterations observed in OA pathology involves quantitative and qualitative change in HA content [44]. Specifically, under the action of reactive oxygen species released by inflamed chondrocytes, both HA amount and MW result consistently reduced [45,46]. Physiologically, HA represents the backbone of the lubrication layer; the loss of its homeostasis has a dramatic impact on the friction coefficient between the two mating cartilage surfaces. The compromission of the boundary lubrication layer determines an increase in friction, resulting in a higher cartilage shear strain. Under this condition, chondrocyte upregulates the production of cartilage-degrading enzymes via mechanotransduction [47]. More in details, chondrocytes start producing matrix metalloproteinases (MMPs) and a specific disintegrin with MMPs’ functions and a thrombospondin motifs (ADAMTS), which are capable of degradingtype II collagen and aggrecan, respectively [48,49]. At the same time, when the proper lubrication is lost and inflammation starts, macrophages migrate to the joint site and, together with chondrocytes, try restoring the homeostasis responding to these stimuli, contributing to the secretion of additional cartilage-degrading enzymes [50,51]. As in other cases, the attempt to resolve tissue inflammation might end in additional tissue damage. In this scenario, the mechanical and proteolytic disruption of the ECM produces a series of fragments. The presence of these debris might have a double detrimental effect: on one side, it might furtherly alter the lubrication properties of the SF; on the other, it might activate macrophages to produce other inflammatory mediators, MMPs and ADAMTS, contributing to ECM damage [52]. In the light of this, researchers have focused on alleviating and treating OA by restoring the lubrication properties of healthy cartilage. In the following sections, we will explore the benefits and the limits of lubricants applied in OA, starting from the natural ones, regularly used in medical treatments, then moving to those more innovative and in experimentation [49].

## 5. Natural Lubricants Based on HA and Its Hydrogel-Formulated Derivatives

As mentioned above, due to the current lack of clinically available disease-modifying OA drugs, restoring the lubrication is the most promising strategy for limiting cartilage erosion and inflammation. The clinically approved therapeutic approach involving natural compounds is based on HA visco-supplementation [53]. This strategy, consisting of IA injections of exogenous HA, replenishes the notable HA decrease observed in OA pathology. This intervention, when applied in the early stages of the disease, has the potential to limit cartilage erosion and inflammation, and to ameliorate pain [53,54]. In 1993, Balazs and Denlinger were the first to demonstrate that the beneficial effects of IA-injected HA were mainly connected with restoration of SF rheological properties [55]. Also, the remarkable safety profile of HA has facilitated the clinical translation of this product, which received approval from the Food and Drug Administration (FDA) in the US in 1997. Years later, it has been demonstrated that the therapeutic benefit of HA IA is attributed not only to its capability of restoring lubrication but also to its chondroprotective biochemical functions, exerted through its multifaceted action on various pathways, receptors, and enzymes (Table 2) [17]. In this regard, Yasuda et al. found that HA suppresses the production of prostaglandin E2 via downregulation of nuclear factor-κB in human macrophages [56]. In line with this finding, Sasaki et al. demonstrated that HA inhibits the expression and production of MMP-1 and MMP-3 in IL-1β-stimulated human synovial cells [57].

The several pharmacological effects HA can produce are anyway limited to its residency time in the articular cavity and its overall half-life. For this reason, the therapeutic effects are limited in time, requiring a relatively high frequency of treatment to achieve a longer effect. To understand if the use of HA with different MW could be beneficial to reduce the frequency of treatments while maintaining its functions, hydrogel-formulated derivatives started being developed. Hydrogels are three-dimensional networks of cross-linked polymer chains that broadly swell in water while maintaining their shape and mechanical strength. As a result of years of research on this specific topic, a series of commercial hydrogel-based visco-supplement formulations for the treatment of OA were developed worldwide [58]. Wang et al. demonstrated that high-MW HA, with supposed longer residency time, was nevertheless able to downregulate the expression of OA-associated cytokines and enzymes in FLS from patients with early OA [59]. In line with the aim of slowing down its clearance and testingtheir bio-active features, other modifications on this macromolecule were also tested, such as the introduction of distinct levels of intramolecular cross-linking. All the FDA-approved HA-based products with linear or cross-linked structures for the treatment of OA are collected in Table 3.

As expected, given the unique features of the specific product in terms of MW and structure (linear or cross-linked), the doses and the treatment regimen vary considerably, based on the specific HA. To further dissect the influence of MW on the therapeutic potential of HA, it is important to mention the preclinical study of Smith and Ghosh. In this manuscript, authors demonstrated that the treatment of human synovial fibroblasts (derived from OA patients) with exogenous HA stimulates the synthesis of new endogenous HA from the cells. This effect was found to be dependent on the MW of the applied exogenous HA [60]. The most significant increase in endogenous HA synthesis from the cells was observed when the treatment was performed with exogenous HA having a MW ranging between 500 and 4700 kDa. Treatments performed with exogenous HA with low MW (below 500 kDa) exhibited no significant effect, while those performed with high HA MW (above 4700 kDa) demonstrated a reduced impact on HA synthesis. A changing binding affinity to HA receptors on synovial fibroblasts might explain the different efficacy of the diverse MW of the exogenous HA molecules tested. Also, in vivo investigations conducted in preclinical animal models of OA showed that the 500 and 1000 kDa HA injections resulted in a partial restoration of synovial cell metabolism and the normalization of HA biosynthesis [60,61]. Despite the clear correlation between HA MW and its therapeutic activity (found both in vitro and in vivo), the same findings could not be consistently replicated in clinical settings. The contradictory results, obtained in different studies, did not allow so far to find a consensus regarding the advantages of IA injection of low- vs. high-MW HA [62]. In authors’ opinion this might anyway depend on the broad pathological variability OA patients have shown, which would require widening the analyzed population and, at the same time, subtyping different phenotypes underlying shared pathobiological features. 

Besides clinical product, other modifications of the native HA molecule were proposed by researchers to further prolong its half-life and enhance its lubrication features in osteoarthritic joints [63]. For example, dopamine-conjugated HA was designed for its bio-adhesive properties and found applications as a tissue adhesive and anti-fouling coating on biological devices. HA–dopamine was tested ex vivo as cartilage lubricant by using a bovine cartilage model, showing a better adsorption onto the cartilage surface in comparison with non-modified HA, while also resulting in a more efficient boundary lubrication. In another work, Xie et al. developed two types of modified HA backbones, one with sulfonate-rich polymers resembling lubricin (poly-2-acrylamide-2-methylpropanesulfonic (PAMPS)) and another with phosphocholine-rich polymers resembling PL (poly-2-methacryloyloxyethyl phosphoryl choline (PMPC)). These two biomimetic brush-like nanofibers, named HA/PA and HA/PM, exhibited a strong affinity for cartilage proteins. Both were able to form a lubricating layer on damaged human cartilage, resulting in reducing friction to the levels measured in healthy cartilage. Moreover, their IA injection into rats with surgically induced OA resulted in cartilage regeneration and an overall amelioration of OA within 8 weeks [64]. Another interesting approach involved the use of intermediate adhesive that can guide injected HA to the cartilage surface. In view of this, Faust et al. developed HABP2-8-arm PEG-COLBP, a heterobifunctional poly(ethylene glycol) (PEG) conjugated with HA-binding and collagen-binding peptide. The peptide polymer was tested in vivo in an anterior cruciate ligament transection murine model of post-traumatic OA. The treatment lowered Interleukin (IL)-6, IL-1β, and MMP13 levels, increased aggrecan expression, and also reduced pain (as measured by incapacitance and hotplate testing) and cartilage degeneration (as measured by OARSI scoring) [65].

## 6. Innovative Hydrogel-Based Strategies for Lubrication and Drug Delivery

In addition to HA-based hydrogels, the recent advancements in material science allowed scientists to design and develop a series of preclinical hydrogel products with enriched lubricant features. This innovative therapeutic approach counts two major classes: (I) bulk hydrogel with structural modification allowing for superior lubricant and cushioning features, some also applicable as cartilage substitute; (II) hydrogel particles composed of polymer chains cross-linked in small dimensions dispersed in colloidal solutions [10]. In the next sections, we will present in detail the most recent advancements in this research niche, moving from innovative hydrogel to micro- and nano-gels.

a. Non-HA-based Hydrogels with superior Lubricant features:

The development of non-HA-based HA hydrogels is relatively recent and does not count yet for clinically approved products. This explorative field led to the development of several products which are worth mentioning, considering their potential future development and application. Zhou’s group established, for example, a series of stiff, robust, and wear-resistant hydrogels by binding hydrophilic polyelectrolyte to the subsurface of another hydrogel with higher stiffness, obtaining a bilayer structure. The morphological and the mechanical characteristics of the product were studied in vitro, with a focus on the kinetic growth and the friction behavior. Results demonstrated that the hydrogels had cartilage-like characteristics, with low friction and high strength [66,67,68]. Another innovative product was presented in the study conducted by Zhao et al. Authors produced a hydrogel composed of one lubrication phase and one load-bearing phase, obtained by chemically binding a thick, highly hydrophilic poly (3-sulfopropyl methacrylate potassium) layer (lubrication phase) onto the subsurface of a three-dimensional elastomer scaffold-hydrogel matrix (load-bearing phase). The lubrication performances of the hydrogel were investigated by using a conventional reciprocating tribology instrument, testing different conditions. The obtained outcomes showed that the highly hydrated lubrication phase allowed to record friction coefficients at a given load (1 N) or dynamic loads (from 0.2 N to 4 N) under a wide range of shear frequencies [69]. It was also demonstrated that the load-bearing phase served as structural support to disperse the applied stress, being able to store elastic strain energy via elastic deformation. This particular feature revealed advanced load-bearing performances. Therefore, this work pointed out that robust, load-bearing bulk, combined with good surface lubrication could represent a promising approach for developing cartilage-like hydrogels. Another strategy to increase lubrication features of the hydrogels is the enrichment with lipids, usually formulated as vesicles and acting as reservoirs, thus forming a thin lubricating layer on the hydrogel’s surface. In this regard, Lin et al. conducted an interesting study on the fabrication of self-lubricating hydrogels by incorporating 1,2-dimyristoyl-sn-glycero-3-phosphocholine (DMPC) and hydrogenated soy phosphatidylcholine (HSPC) in the form of multilamellar vesicles (MLVs) into the poly(hydroxyethyl methacrylate) (pHEMA) hydrogel. Frictional characterization of all hydrogels was performed with a back-and-forth mode with the aim to estimate the friction between the hydrogel and a polished stainless-steel surface at 25 °C and 37 °C, over different loads or contact pressures. Results showed that hydrogels incorporating the MLVs displayed a reduction in friction and water loss of about 80 to 99.3% when compared with the lipid-free pHEMA hydrogels. The authors demonstrated that the reduction in friction and water loss was related to the continuous self-renewal of the molecularly thin lipid-based boundary layer between the sliding surfaces (hydrogel and polished stainless surfaces). In other words, as the friction abraded the hydrogel, the incorporated lipids were progressively exposed [70]. A similar strategy was developed by Feng and colleagues, who formulated super-lubricated hydrogels designed for natural joints and suitable in cartilage replacement. Briefly, to enhance its lubrication ability, the composition of the PMS hydrogel (obtained via the free-radical polymerization of poly(sulfobetaine methacrylate)-PSBMA- and 2-methacryloyloxyethyl phosphorylcholine -MPC-) was enriched with HA and HSPC in the form of MLVs or single unilamellar vesicles (SUVs). The obtained super-lubricated hydrogels (PMS-HSPC(SUV)-HA and PMS-HSPC(MLV)-HA) were carefully characterized. In particular, friction experiments were performed using a universal micro tribometer, with a reciprocating mode of ball-on-disk. Results demonstrated that, under a load of 1 N, PMS-HSPC(MLV)-HA and PMS-HSPC(SUV)-HA μ was 0.0089 ± 0.0035 and 0.0052 ± 0.0019, respectively. Revealing PMS-HSPC(SUV)-HA reduced by 85% the results obtained with the PMS hydrogel (μ = 0.0322 ± 0.0069). The super-lubrication state was constant also with the rehydrated gel or having different experimental conditions such as sliding with different materials and velocities. This behavior was attributed to the synergistic lubrication effect between lipids and HA after the formation of uniformly arranged liposomes around HA structure [71]. Another interesting example is the research conducted by Xiao et al., who worked on a copolymer consisting of HEMA and N, N-dimethylacrylamide (DMAA) (p(HEMA-co-DMAA)) incorporated with DMPC-MLV which could be applied as soft tissue substitute for articular cartilage. The obtained lipid–hydrogel showed remarkable properties in stiffness and load-bearing. Moreover, when the molar ratio of HEMA to DMAA was 3:1, the maximum compressive modulus and the maximum compressive strength were reached at 4.7 and 5.8 MPa, respectively. As for the friction characterization, when the hydrogel was under a load of 5 N, the friction coefficient was as low as 0.026. However, when the applied load was increased to 30 N, the friction coefficient increased to ~0.2, limiting its application as a cartilage substitute, given the high pressure in the knee joint [72]. Based on the examples reported above, authors believe that the development of innovative hydrogels to achieve excellent lubrication properties could represent a keystone to obtain formulations able to reproduce the cartilage characteristics and guarantee, at the same time, low friction coefficient and excellent mechanical and structural support.

b. Micro-gels and Nano-gels

Micro-gels and nano-gels, also known as hydrogel MPs and NPs, are novel formulations of hydrogels composed of polymer chains typically cross-linked in spheres of diverse dimensions or in other shapes. Their classification depends on size: micro-gels are larger than 1 µm, and nano-gels are smaller than 1 µm [73]. Both these particle-based classes of formulations possess a significantly enhanced injectability, flowability, and flexibility, which allow for their effective IA-dispersion during joint loading [74]. The lubricating capacity of these hydrogel systems depends on the characteristics of the biomaterial utilized in formulating them [75]. They are designed for having an extended residency time into the joint and they can be loaded with drugs, employing a two-pronged approach [73]. This capability is particularly important since OA patients, in addition to IA injections of biolubricants, are often prescribed with orally administered Non-Steroidal Anti-Inflammatory Drug (NSAID) or IA-injected corticosteroids, to reduce pain and inflammation. Micro- and nano-gels offer the chance to co-administer anti-inflammatory molecules which can be released over a long period of time [76]. In this context, it is clear that different sizes and physio-chemical features can be exploited for different purposes. For example, the larger particles are in general more oriented on acting on the matrix rather than on cell, exhibit a larger resistance to lymphatic and vascular drainage, and generally grant better lubrication and a prolonged release [77]. Conversely, those ones smaller in size are more easily internalized into superficial chondrocytes or synoviocytes, making them particularly suitable for conditions requiring both a degree of lubrication and enhanced cellular delivery of therapeutic agents [78]. In the following sections, we will explore the most recent advancements in hydrogel-based MPs and NPs by reporting some examples. The cited works are also summarized into separate tables (Table 4 and Table 5) compiling the main features of the particles and a summary of the outcomes.

In a notable study, Han et al. designed some lubricating microspheres named GelMA@DMA-MPC. These particles were fabricated by dip-coating a self-adhesive copolymer (DMA-MPC: dopamine methacrylamide-2-methacryloyloxyethylphosphorylcholine) onto the surface of photo-cross-linked gelatin methacrylate (GelMA) hydrogel microspheres loaded with diclofenac sodium (DS). The self-adhesive features of the copolymer were granted by the presence of dopamine methacrylamide, which possesses a well-established cartilage adhesion function. In addition, the presence of 2-methacryloyloxyethyl phosphorylcholine sodium (with zwitterionic charges) promotes hydration-lubrication processes. GelMA@DMA-MPC showed a mean size of approximately 150 μm; tribological and drug release tests confirmed enhanced lubrication and sustained drug release of DS over 14 days. In particular, tribological experiments using a pin-on-disk friction pair assessed the lubrication properties of GelMA and GelMA@DMA-MPC: both GelMA formulations demonstrated a lower µ compared to PBS (µ = 0.027). However, GelMA@DMA-MPC outperformed GelMA by further reducing the µ through hydration-lubrication mechanisms provided by the zwitterionic phosphocholine groups in the copolymers (µ = 0.019). To evaluate the therapeutic effect of these biomimetic formulations in vivo, a DMM rat model of OA was used. One week post-surgery, rats received IA injections of PBS, GelMA, GelMA@DMA-MPC, or GelMA@DMA-MPC@DS every two weeks for 8 weeks. Results showed that the GelMA@DMA-MPC@DS group exhibited the greatest inhibition of OA progression, with notable improvements in GAG deposition, cartilage morphology, lesion depth, OARSI score, and cartilage matrix depletion. Considering these findings, GelMA@DMA-MPC@DS offers promising therapeutic effect for early OA [79]. Similarly, Yang and colleagues fabricated GelMA photo-cross-linkable microspheres with controllable particle size (~100 µm) using a microfluidic emulsion method (denoted as MGS). These MPs were modified on the surface via one-step dip-coating approach with a novel copolymer (dopamine methacrylamide-sulfobetaine methacrylate: DMA-SBMA) containing PSBMA brush and dopamine. Thanks to their porous structure, MGS@DMA-SBMA were loaded with DS (MGS@DMA-SBMA@DS), via dynamic physical absorption, forming the final drug-loaded and lubricating MP which was then tested in vitro and in vivo. Authors showed that the incorporation of PSBMA brushes endowed the MGSs with enhanced lubricating properties and sustained drug release ability. Lubrication properties were measured using a universal materials tester (UMT-3, Bruker Nano Inc., Berlin, Germany). MGSs exhibited a 25% µ reduction compared to PBS (µ = 0.032). Surface grafting with DMA-SBMA copolymer further decreased µ by 11%, due to the stable hydration layer formed around the zwitterionic charges (–N+(CH_3_)_2_– and –SO_3_−). These microparticles also exhibited excellent biocompatibility and were able to protect chondrocytes against inflammatory factor-induced degeneration in vitro. To assess the therapeutic effects of these hydrogel MPs in vivo, a DMM rat model of OA was used. One week post-surgery, rats were divided into four groups and injected intra-articularly every two weeks with 100 µL of PBS, MGS, MGS@DMA-SBMA, or MGS@DMA-SBMA@DS. Results showed that drug-loaded super-lubricated MGSs had the most pronounced therapeutic effect on OA and significantly reduced osteophyte formation and cartilage degradation [80]. Along the same lines, Lei et al. synthesized rapamycin-loaded hydrogenated soy phosphatidylcholine (HSPC) liposomes incorporated into HA-based hydrogel microspheres (RAPA@Lipo@HMs). Both microfluidic technology and photopolymerization processes were used for the fabrication. These MPs presented a size of ~200 µm and formed self-renewable hydration layers, improving lubrication through a smooth rolling mechanism. Lubrication performances of PBS, liposome-free hydrogels (HM), and worn Lipo@HMs were evaluated under the same conditions using a universal materials tester (UMT-3, Bruker Nano Inc., Berlin, Germany). The calculation of µ revealed a value equal to 0.06 for PBS, while HMs and Lipo@HMs were able to reduce µ to 0.04 and 0.03, respectively. Additionally, the release of autophagy activator rapamycin loaded into cationic liposomes within the HMs allowed them to target negatively charged cartilage through electrostatic interactions, thereby supporting cellular homeostasis by boosting autophagy [81]. To assess whether RAPA@Lipo@HMs could alleviate joint wear and reduce osteoarthritic degeneration in vivo, a DMM rat model of OA was used. Data demonstrated that this novel formulation significantly alleviated joint wear and delayed the progression of early OA. The systems so far described in this MP section all presented a spherical shape, but it is important to underline that hydrogel MPs can also present alternative geometries. One example of a non-spherical MP is offered by the squared Poly(lactic-co-glycolic acid)-based MP (µ-plates), which were proposed for the treatment of OA. This novel formulation combines a series of features, since they can: (i) be IA-injected; (ii) deliver small molecules and nanoparticles; (iii) act as a drug depot: (iv) enable a sustained release of payloads; and also (v) give mechanical support to the joint, thus paving the way to a unique and innovative therapy for the OA [82,83].

**Table 4:** Hydrogel MP-based lubricant systems.

Name and Microparticle Structure	Material	Size	Outcomes	Ref.
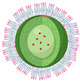 GelMA@DMA-MPC	GelMA microspheres coated with DMA-MPC and loaded with DC	150 µm	GelMA@DMA-MPC showed enhanced lubrication and sustained drug release of DC. Injected into rat knee joints with osteoarthritis, they showed significant therapeutic effects.	[79]
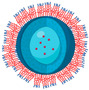 MGS@DMA-SBMA	GelMA microspherese coated with DMA-SBMA and loaded with DC	100 µm	MGS@DMA-SBMA demonstrated improved lubrication abilities and provided chondroprotection both in vitro and in vivo in an OA rat model.	[80]
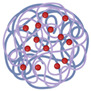 RAPA@Lipo@HMS	RAPA-liposome– incorporating HA–based HMs	200 µm	RAPA@Lipo@HMs enhanced joint lubrication with a smooth rolling mechanism and continuous exposure of liposomes on the surface, forming self-renewing hydration layers through friction.	[81]
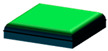 µPlate	PLGA	20 µm	Drug depot with sustained release; mechanical support to the joint; small molecules delivery.	[82,83]

As mentioned before, together with micro-gel formulations, also a series of hydrogel-based nano-gels with lubricant and drug delivery properties were recently proposed for being tested in preclinical studies [66,84]. Also, these formulations are meant to be injected in the joint cavity, but, with respect to MP, their contact surfaces are more extended; through small-gap infiltration, NPs can efficiently modify joint tribology by offering a protective layer also to those areas which are more difficult to reach by MP. In other words, they behave as interposed ball bearings between surfaces [85]. We collected some of the most recent examples of nano-gels in the following text and summarized their main features in Table 5.

In a remarkable work, Liu et al. utilized negatively charged poly(3-sulfopropyl methacrylate potassium salt) (PSPMK) brushes grafted onto poly(N-isopropylacrylamide) (PNIPAAm) to generate nano-gels (SB-g-NBrMGs). These ~500 nm nano-gels incorporated PNIPAAm cores for thermosensitive drug release and were grafted with PSPMK brushes, endowing them with a biomimetic polyelectrolyte structure for effective hydration lubrication. The resulting hairy nano-gels showed notable tribological properties and temperature-triggered drug release ability. Importantly, they maintained an ultra-low friction coefficient, even at elevated temperatures, surpassing the performance of traditional PNIPAAm micro-gels. The broad temperature range of effective lubrication is advantageous for in vivo applications and could furnish a potential new system for treating arthritis in clinical settings [86]. By producing smaller NPs (~200 nm), Maudens et al. introduced for the first time the concept of self-lubricating nano-ball bearings as a new strategy to improve joint lubrication. In this study, authors developed a new injectable HA-PNIPAM nano-hydrogel by conjugating HA to a thermo-responsive polymer, thus allowing the spontaneous formation of nanosized structures at body temperature. The formulations were investigated for their injectability, sensitivity to enzymatic degradation, and cytocompatibility. By performing subcutaneous and IA injections to healthy mice and a murine OA model, authors found these NP to be able to remain in the joint up to 21 days. In addition, HA-PNIPAM resulted efficacious in protecting the cartilage, in preserving the epiphysis thickness, and in reducing the expression level of VEGF, IL-1β, and TNF-α [87]. A similar work was conducted by Zhang et al., who studied thermo-sensitive dual-functional nanospheres, based on PNIPAM and poly [N-isopropylacrylamide-2-methacryloyloxyethyl phosphorylcholine] (PNIPAM-PMPC), synthesized through emulsion polymerization. The great lubrication properties (µ = 0.02) of PNIPAM-PMPC, analyzed by using a pin-on-disk universal material tester, were attributed to the interaction between the water dipole and the zwitterionic headgroups of the PMPC. Therefore, the nanospheres were able to support high pressures, behaving as a fluid under shear with a significant decrease in interfacial friction. Moreover, the nanospheres could efficiently load the anti-inflammatory drug DS and release it following a thermo-sensitive fashion [88]. Yang et al. investigated a chitosan NP-based biomimetic lubricant, obtained by chemically binding hydrophilic sulfonic acid groups on particle surface. Due to the resulting negative surface charge, the formulation resulted in a notably low µ (0.01, measured by using a conventional reciprocating tribometer at a ball-on-disk mode). These low friction levels were also maintained under a wide range of loads, frequencies, and concentrations. Moreover, the NPs showed a good ability to deliver molecules such as aspirin [89]. In a different study, reported by Ren et al., self-assembled nanosized lubricated additives named CBPXGSB1/5 were obtained. More specifically, they synthetized a modified polymer derived from the xanthan gum, grafted by PSBMA, and then conjugated it with a Collagen II-Binding peptide. Outcomes highlighted that the hydrated effect of PSBMA side chains provided CBPXGSB1/5 with suitable lubrication property [90]. Anilkumar et al. reported an innovative lubricant composed of soft nanometric single hyperbranched glycerol polymers with MW > 1,000,000 (mega HPGs). Among the different features, they showed high water solubility and low intrinsic viscosity, thus acting as interposed ball bearings between both hard and soft surfaces to reduce µ, as shown in [91].

**Table 5:** Hydrogel NP recently proposed for the treatment of OA.

Name and Nanoparticle Structure	Material	Size	Outcomes	Ref.
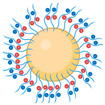 SB-g-NBrMGs	PSPMK brushes-grafted PNIPAAm microgel	~500 nm	These hairy microgels showed notable tribological properties and temperature-triggered drug release ability.	[86]
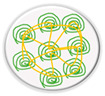 HA-PNIPAM	HA-grafted PNIPAM	~200 nm	Improved injectability, sensitivity to enzymatic degradation, and cytocompatibility. Prolonged joint retention joint, cartilage protection and reduction of pro-inflammatory cytokines.	[87]
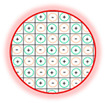 PNIPAM-PMPC	PNIPAM PMPC	~200 nm	Resistant to high pressure, efficient DDS for DC, able to control the thermo-sensitive drug release.	[88]
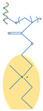 Chitosan NP	Chitosan NP grafted with hydrophilic sulfonic acid groups.	~160 nm	Low friction coefficient (µ = 0.01) and valuable DDS.	[89]
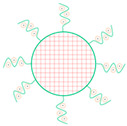 PSBMA-CBPXGSB1/5	Xanthan gum PSBMA Collagen II-Binding peptide	~280 nm	Good lubrication proprieties.	[90]
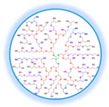 mega HPGs	Hyper-branched glycerol polymers	~20–50 nm	High water solubility and low intrinsic viscosity.	[91]

In the context of nanosized-based hydrogels, the contribution of mesoporous silica NPs (MSNs) needs to be mentioned considering the extensive research on the modifications aimed to enhance lubrication and the drug delivery features of this material. Some examples of this group of therapeutics are collected in Table 6. Among them, it is worth mentioning the interesting study by Wan and colleagues, who reported the synthesis of MSNs grafted by photopolymerization with the zwitterionic polymer: 3-[dimethyl-[2-(2-methylprop-2-enoyloxy)ethyl]azaniumyl]-propane-1-sulfonate polymer (pSBMA) to obtain MSNs@pSBMA. Thanks to the hydration-lubrication mechanism, these NPs showed improved lubrication properties (μ = 0.045) and a 80% µ reduction when compared to MSNs (μ = 0.221). These NPs were designed to treat early-stage OA and can count on a synergistic therapy of lubrication restoration and on the local release of drugs. In this study, Rhodamine B was used as model drug, to be loaded at different concentrations in the NPs. The release studies demonstrated that the MSNs@pSBMA allowed a sustained release of the cargo [92]. A different approach was used by Liu et al., who produced core/shell charged polymer brush-grafted hollow MSNs (PSPMA-g-HSNPs-0.5%; 0.5% is the solid content), showing good drug loading-release capability and a strong lubricating effect (μ = 0.173). In this specific case, the combination of (i) the hydration-lubrication mechanisms favored by the hydrated layers of the PSPMA brushes and (ii) the rolling lubrication due to the free rolling of the NP were both crucial for reducing friction [93]. In a different work, the effect of a PL coating on MSNs was studied. Sun and colleagues designed and tested MSNs@lip, for the treatment of early-stage OA. They demonstrated that the presence of PL reduced µ to 0.05 when compared to uncoated MSN (μ = 0.2). The group also found this was due to the increase in hydration-lubrication processes [94]. A similar functionalization was optimized by Chen et al., who proposed poly PMPC-grafted MSNs (MSNs-NH2@PMPC) via photopolymerization for the treatment of both early- and late-stage OA. Indeed, these NPs enhanced the articular cartilage lubrication (μ = 0.015) due to the combined effect of hydration-lubrication and boundary lubrication mechanisms (early-stage OA treatment). Moreover, Chen’s group was also able to load MSNs-NH2@PMPC with the DS by controlling its release, a good candidate formulation for the late-stage OA treatment [95]. In a different work, Zhao et al. decorated the surface of MSNs by supramolecular interaction between azobenzene (AZO) and β-cyclodextrin (CD)-modified poly(2-methacryloyloxyethyl phosphorylcholine) (CD-PMPC), obtaining a dual-functional biodegradable NP, suitable for both lubrication and drug delivery. This nano-system is able to: (i) allow for considerable lubrication functions (μ < 0.04) thanks to the hydrated layer around the zwitterionic charged groups in CD-PMPC; (ii) release DC in response to visible light acting on azobenzene isomerization. bMSNs-AZO/CD-PMPC was not tested yet in vivo, but in vitro demonstrating its good cell compatibility and promising anti-inflammation activity [96]. Another notable approach was used by Yan et al., who described lubricated NP inspired by the natural structure of the Euryale ferox seed, namely PSPMK-grafted MSNs (MSNs-NH2@PSPMK) obtained via a one-step photopolymerization method. These NPs showed great loading capability and controlled release of DS, and, due to the grafted PSPMK polyelectrolyte polymer (brush-like), they also provided enhanced lubrication in the joint (µ~0.065). In vitro and in vivo experiments showed that the DS-loaded MSNs-NH2@PSPMK NPs efficiently inhibit chondrocyte degeneration and slow the course of OA [97].

A wide vision on these studies highlights the concept that a series of therapeutic chances arise from the use of MPs and NPs considering the possibility of: (i) functionalizing them by using bio-adhesive copolymers to increase their adhesion to articular tissue; (ii) modifying them with zwitterionic moieties to increase the hydration lubrication; (iii) decorating them with PL to improve their lubrication performance; (iv) modifying them to make them more resistant to degradation; (v) loading them with therapeutic agents showing a controlled and sustained release. This high grade of versatility and the notable performances in the mechanical and lubrication support provided to the joint encourage scientists to combine different methods and approaches. 

Of course, extensive research needs to be dedicated: (i) to furtherly characterize the specific physicochemical properties of bio-adhesive materials on their performance; (ii) to systematically optimize the balance between lubrication, drug release kinetics, and material degradation resistance; (iii) to explore the impact of particle size and shape. Despite the partial lack of this information, we believe the versatility of these novel systems could likely represent the basis for more efficacious OA treatments to be validated in clinical settings.

## 7. Innovative Liposome-Based Strategies for Drug Delivery and Lubrication

Besides hydrogel-based formulation, liposomes were also tested for the treatment of OA. Liposomes are sphere-shaped vesicles having one or more PL bilayers surrounding an aqueous core. They are currently used as DDSs to encapsulate hydrophilic agents in the aqueous core and/or hydrophobic ones within the lipid bilayers [98,99]. Depending on fabrication and composition, liposomes can be formulated in various sizes, ranging from 20 nm to about 5 μm [100]. In the context of OA therapy, liposomes were firstly tested as drug depots aimed to reduce the frequency of anti-inflammatory drugs’ injection; Table 7 reports the liposomal formulation used as DDSs cited in this manuscript. More recently also their mechanical functions as joint lubricants started to be studied. In view of this, their application for the treatment of OA could benefit from this double function [101,102]. The following section reports recent preclinical and clinical studies related to these two approaches. Taiwan Liposome Company developed TLC599, a ~130 nm clinical liposomal formulation of dexamethasone sodium phosphate (DSP). The liposome is composed of: 67.5% 1,2-dioleoyl-sn-glycero-3-phosphatidylcholine (DOPC), 7.5% l,2-dioleoyl-sn-glycero-3-phosphoglycerol (DOPG), and 25% cholesterol; 12 mg of DSP per 100 μmol of PL were encapsulated [103]. TLC599 showed a long-lasting profile remaining up to 120 days in synovial joint after a single IA injection in a preclinical dog study. In addition, no significant systemic exposure and accumulation of DSP and dexamethasone (DEX) in dog plasma was observed following multiple-dose administration of TLC599 [104]. Thanks to these promising results, this formulation has recently reached the clinical stage being applied in two clinical trials. The trials confirmed the TCC599 long-lasting permanence also in human synovial joints, while granting for a minimal systemic exposure. In a clinical study run on patients with Kellgren–Lawrence grade 2 or 3 for knee OA severity, the treatment was well tolerated and reduced pain for up to 24 weeks. Taking also into account formulation safety and efficacy, its advancement to clinical stages is very probable. Details of the specific studies can be found here: NCT02803307 and NCT03005873 [105]. Beside DSP, also other drugs for the treatment of OA were loaded into liposomes and tested in preclinical studies. For example, a collagen type I/dipalmitoyl phosphatidylethanolamine (DPPE) liposome system (collagomer) loaded with the NSAID DC was developed by Elron-Gross et al. [106]. Magnetic Resonance Imaging analyses revealed that the IA administration of DC-collagomer (1 mg kg^−1^ DC) in a rat model of OA significantly reduced inflammation volume (water accumulation) by ~75% in 3 weeks. This result showed a statistically significant difference vs. untreated rats (~50%) and vs. conventionally treated ones (~38%). Although both unloaded collagomers and DC ones decreased inflammation, combining them did not exhibit substantial additional benefits, possibly due to high variability in these groups. In a different study, Corciulo et al used phosphatidylcholine (PC) and cholesterol liposomes to encapsulate a critical autocrine factor for maintenance of cartilage homeostasis: adenosine. An alternative formulation was also generated by loading a selective adenosine A2A receptor agonist: CGS21680. The study demonstrated that both formulations significantly decreased OA cartilage damage in a murine model of obesity-induced OA. In addition, an in vitro experiment on primary chondrocytes (harvested from knees of rats with post-traumatic OA) showed a reduced expression of genes associated with matrix degradation and an upregulation of genes associated with cell proliferation when treated with liposomal A2AR agonist as compared to liposomes alone [107]. In another study, positively charged liposomes (53 mV), composed of PhosphatidylEthanolamine (PE), 1,2-DioleOyl-3-TrimethylAmmonium Propane (DOTAP), and 1,2-dipalmitoyl-sn-glycero-3-phosphoethanolamine (Liss Rhod PE), were instead used for the delivery of mitochondria (MT) to chondrocytes. Recent studies revealed that MT dysfunction might play a significant role in the pathological mechanisms of OA. The in vitro and in vivo results suggested that the chondrogenic expression of inflammatory cytokines decreased while the expression of ECM components increased upon the delivery of MT. This phenomenon indicates that the restoration of functional MT might have a great potential in recovering cell homeostasis in tissues damaged by OA [108]. As anticipated, liposomes were investigated not only as drug release depots but also for their potential to improve biolubrication in OA-damaged cartilage. This hypothesis arose since PLs (commonly used for liposomes formulations) are directly involved in the biolubrication of the synovial articular joints [109,110,111]. An example in this regard is provided by the study of Kawano et al. In this work, the effects of eight consecutive weekly IA administrations of: (i) 2000 kDa HA (high MW), (ii) 800 kDa (medium MW), and (iii) a combination of 2000 kDa HA with 1,2-dipalmitoyl-sn-glycero-3-phosphocholine (DPPC) liposomes were compared in a rabbit OA model. In three distinct experimental cohorts, these treatments were delivered IA in the rabbit model of OA induced by anterior cruciate and medial collateral ligament transaction. Histological analysis of the articular cartilage post-treatment revealed that there was no substantial regeneration of the diseased cartilage in the groups treated solely with HA. However, the combined treatment group (HA + liposomes) exhibited significantly less damage to the cartilage, indicating that for the prevention of articular cartilage degeneration, the addition of liposomes would improve the therapeutic outcome. The addition of liposomes determined a µ reduction to (0.0150 ± 0.0033), showing a significant difference with OA mice (0.0206 ± 0.00649) and with HA-only-treated mice (0.0190 ± 0.00427 for the high MW and 0.0177 ± 0.00712 for the low MW). At the same time, a not-significant difference with healthy CTRL (0.0100 ± 0.00300) was found, revealing that the treatment restored µ level found in healthy mice [112]. In another study, the cartilage-lubricating effect of a variety of PC-based liposomal formulations, including SUV with sizes below 100 nm and MLVs (liposomes with concentric layers of lipids) larger than 800 nm, was investigated using a human-sourced cartilage-on-cartilage apparatus that mimics articular joint [113]. It was observed that MLVs demonstrated superior lubricating properties compared to SUVs. The results of the study also suggested that the most effective lubrication was achieved when using DMPC with a transition temperature slightly below body temperature. The study emphasized the importance of hydration and high compressibility, as essential conditions for achieving effective lubrication. Following the same approach, Klein et al. examined SUV composed of hydrogenated L-α-PC (HSPC) lipids, with a size of 65 nm, as a potential boundary-lubricating agent. Surface force balance studies were conducted to investigate the normal and shear forces under high loading conditions, revealing extremely low coefficients of friction. The researchers proposed that certain liposomes may generate close-packed boundary layers on surfaces under water that lead to a striking reduction in the sliding friction. At the highest pressures, in fact, the surfaces exhibited a “hard-wall” repulsion, with a separation of approximately 21 ± 2 nm, corresponding to four bilayers derived from the two flattened liposome layers. They also found out that when the highly hydrated outer phosphocholine layers are coated with PEG, a µ increase is registered: (0.0004–0.00002 without PEG vs. 0.1075 ± 0.084 with PEG) [114]. In a recent publication, Zhong et al. used liposomes for both purposes, as DDSs for the drug meloxicam and as lubricant. The study demonstrated that the IA injection in rat mandibular ramus’s outer surface reduced the ECM degeneration and the synthesis of inflammatory factors, while protecting the cartilage from its progressive wear due to the lubrication potential of the formulation [115]. 

In conclusion, in this paragraph, we reported studies in which liposomes were loaded with different kinds of payload: (i) commonly used anti-inflammatory drugs; (ii) bioactive agent as A2A receptor agonist; and (iii) mitochondria. An overall look at the reported articles indicates that liposomes are particularly suitable for the development of new formulations to treat OA considering (i) their versatility in encapsulating different payloads; (ii) their natural biolubrication features; (iii) the chance to easily improve their performance by modifying their components; (iv) their easy applicability, scalability, and their common use in clinic. In addition, liposomes can be loaded into bulk hydrogel or MPs, generating a hierarchical platform exploiting the advantages of liposomes and those of MPs.

## 8. Conclusions

OA is widely considered the most common degenerative joint disease, mostly affecting elderly people [3]. Considering the increase in the average age of the population, its high incidence currently imposes a substantial economic burden on national healthcare systems [29]. Since putative disease-modifying treatments are still missing, it is relevant to reduce treatment recurrence, both for the benefit of patients and society. The development of more efficient lubricants with slower clearance (from hydrogels to hydrogel MPs and NPs and liposomes) makes a step further in this direction with respect to conventional treatments. Moreover, the possibility to couple anti-inflammatory and lubricant treatment in one single formulation improves patients’ compliance and treatment effectiveness. Overall, the authors can highlight two main important points: (i) the application of the most recent developments in the field of drug delivery to OA; (ii) the discovery that some of these systems can also function as lubricants or can either be designed to have lubricant features. These advancements might also prepare the field for an effective delivery of future drugs treatments which might halt the disease cascade while at the same time act on the mechanics of the joint. In summary, the most effective strategy for counteracting the development of OA might come from systems integrating advanced lubrication features and the sustained release of suitable therapeutic agents. The application of MPs, NPs and liposomes (DDSs) for the treatment of OA might cover this need also considering the possibility to combine some of the reported formulation. The success in this promising path will also depend on researchers’ efforts to investigate the clinical applicability of these novel treatments.

## Figures and Tables

**Table 1:** The name, structure, the properties, the molecular weight, and concentration of natural lubricants in healthy and osteoarthritic joints [44].

Lubricant	Structure	Properties	Concentration (mg/mL)	
			Healthy	Osteoarthritis
Hyaluronic Acid	Repeating disaccharide units of D-glucuronic acid and D-N-acetylglucosamine attached by β (1–4) and β (1–3) glycosidic bonds	HA creates the backbone for PGs of the ECM, protects the cartilage, and blocks the loss of PGs from the cartilage matrix into the synovial space maintaining the physical form of the ECM.	1.6–3.7	1.1–1.9
Lubricin	It is composed by a central mucin-like domain with negatively charged and hydrophilic properties, between two non-glycosylated with positively charged and hydrophobic properties	In the outer superficial zone and at the cartilage surface it interacts with and immobilizes HA	0.305–0.404	0.108–0.183
Phospholipids	Amphiphilic molecules with two hydrophobic diacyl tails and a hydrophilic phosphocholine head group	These exposed groups slide past similar groups from the opposing surface with low friction up to high pressures (100 atm or more) via the hydration lubrication mechanism	0.13–0.15	0.23–0.98

**Table 2:** Physiological and pharmacological effects of HA in the joint [17].

**Physiological effects**
Maintenance of SF viscoelasticity Maintenance of cartilage bio-lubrication Backbone of cartilage ECM
**Pharmacological effects**
Scavenges ROS/RNS and exerts antioxidative effect Exerts anti-inflammatory effect Reduces production of MMPs (MMP-1, MMP-3, and MMP-13) Reduces production and activity of IL-1β, and other pro-inflammatory mediators Inhibits synthesis of PGE2 and bradykinin Regulates fibroblast proliferation Inhibits migration and aggregation of leukocyte and macrophages Enhances synthesis of chondrocytes, HA, and PG Improves viscoelasticity and enhances lubricating potential Improves joint function, mobility, and reduces stiffness

**Table 3:** FDA-approved injectable HA visco-supplementation products [54].

Product	Molecular Weight kDa	Dose (mg)	Frequency	Cross-Linking
Hyalgan	500–730	20 (5 doses)	Weekly	No
Supartz FX	620–1170	25 (5 doses)	Weekly	No
Monovisc	1000–2900	88 (1 dose)	Once	Yes
Orthovisc	1000–2900	30 (3-4 doses)	Weekly	No
Euflexaa	2400–3600	20 (3 doses)	Weekly	No
Synivisc	6000	16 (3 doses)	Weekly	Yes
Durolane	100,000	60 (1 dose)	Once	No
Gel-one	∞	30 (1 dose)	Once	Yes
Synivisc-One	∞	48 (1 dose)	Once	Yes

**Table 6:** Lubricant MSN recently proposed for OA treatment.

Name and Nanoparticle Structure	Material	Size	Outcomes	Ref.
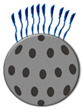 MSNs@pSBM-3	MSNs grafted with pSBMA-3	~100 nm	Improved lubrication properties (μ = 0.045) and reduction of 80% in the coefficient of friction when compared with MSNs (μ = 0.221).	[92]
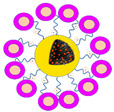 PSPMA-g-HSNPs-0.5%	Core/shell charged polymer brush-grafted hollow MSNs	~739 nm	Controlled drug loading and release; good lubricant effect (μ = 0.173) and reduced coefficient of friction.	[93]
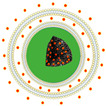 MSNs@lip	Phospholipid-coated MSNs	150–350 nm	Reduced coefficient of friction (μ = 0.05) in comparison with MSNs (μ = 0.2).	[94]
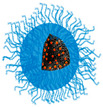 MSNs-NH2@PMPC	PMPC-grafted MSNs	~260 nm	Enhanced lubrication (μ = 0.015) activity.	[95]
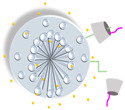 bMSNs-AZO/CD-PMPC	MSNs modified surface with AZO and CD-PMPC	~150 nm	Improved lubrication (μ < 0.04) and enhanced drug release efficiency upon visible light irradiation.	[96]
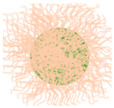 MSNs-NH2@PSPMK	PSPMK-grafted MSNs	~114 nm	Great drug loading capability and controlled release, enhanced lubrication in the joint (µ ~ 0.065), and chondrocytes protection.	[97]

**Table 7:** Liposomal formulations used as DDSs in clinical or preclinical OA studies.

Name/Developer and Liposome Structure	Material	Size	Outcomes	Ref.
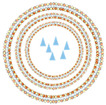 TLC599	DOPC DOPG Cholesterol DSP Dexametason	~150 nm	Long-lasting profile remaining up to 120 days in synovial joint after a single AI in preclinical study in dogs.	[103]
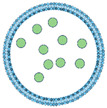 Collagomer	DPPE Collagen I Diclofenac	~10 µm	Significant reduction of inflammation in a rat model of OA.	[104]
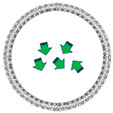 Corciulo, et al.	PC Cholesterol	ND	Significant decrease of OA cartilage damage in a murine model of obesity induced OA.	[105]
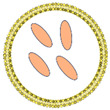 Kim et al.	PE DOTAP Liss Rhod PE Mitochondria	600–700 nm	Significant decrease of inflammatory cytokines; significant increase in ECM components expression.	[106]
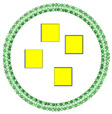 Zhong et al.	HSPC DSPE-PEG2000 Cholesterol Meloxicam	110–125 nm *	Reduced ECM degeneration and reduced synthesis of inflammatory factors in mandibular ramus of rats.	[115]

* for the active loading formulation.

## Abbreviations

AZO	azobenzene
CD-PMPC	β-cyclodextrin (CD)-modified poly(2-methacryloyloxyethyl phosphorylcholine)
DC	dicolfenac
DDS	drug delivery systems
DEX	dexamethasone
DMA-MPC	dopamine methacrylamide-2-methacryloyloxyethylphosphorylcholine
DMA-SBMA-pSBMA	dopamine methacrylamide-sulfobetaine methacrylate
DMAA	HEMA and N, N-dimethylacrylamide
DMM	destabilization of the medial meniscus
DMPC	1,2-dimyristoyl-sn-glycero-3-phosphocholine
DOPC	dioleoyl-sn-glycero-3-phosphatidylcholine
DOPG	l,2-dioleoyl-sn-glycero-3-phosphoglycerol
DOTAP	1,2-DioleOyl-3-TrimethylAmmonium Propane
DPPC	1,2-dipalmitoyl-sn-glycero-3-phosphocholine
DPPE	dipalmitoyl phosphatidylethanolamine
DS	diclofenac sodium
DSP	dexamethasone sodium phosphate
ECM	extracellular matrix
FDA	Food and Drug Administration
FLS	type B fibroblast-like synoviocytes
GAG	glycosaminoglycans
GelMA	gelatin methacryalate
HA	hyaluronic acid
HA-PNIPAM	HA-poly(N-isopropylacrylamide)
HPG	hyperbranched glycerol polymers
HSPC	hydrogenated soy phosphatidylcholine
IA	intra-articular
IL	interleukin
Liss Rhod PE	1,2-dipalmitoyl-sn-glycero-3-phosphoethanolamine
MLS	macrophage-like synoviocytes
MLV	multilamellar vesicles
MMPs	matrix metalloproteinases
MP	microparticles
MSNs	mesoporous silica NP
MSNs-NH2@PMPC	poly(2-methacryloyloxyethyl phosphorylcholine) (PMPC)-grafted MSNs
MSNs-NH2@PSPMK	poly(2-methacryloyloxyethyl phosphorylcholine) (PSPMK)-grafted MSNs
MT	mitochondria
MW	molecular weight
NP	nanoparticles
NSAID	Non-Steroidal Anti-Inflammatory Drug
OA	osteoarthritis
OARSI	Osteoarthritis Research Society International
PAMPS	poly-2-acrylamide-2-methylpropanesulfonic
PE	phosphatidylEthanolamine
PEG	poly(ethylene glycol)
PGs	proteoglycans
PG	prostaglandin
pHEMA	poly(hydroxyethylmethacrylate)
PL	phospholipids
PLGA	Poly lactic-co-glycolic acid
PMS	poly(2-methacryloyloxyethyl phosphorylcholine) (MPC)-co-poly(sulfobetaine methacrylate) (SBMA) hydrogel
PMPC	poly-2-methacryloyloxyethyl phosphoryl choline
PNIPAAm	poly(3-sulfopropyl methacrylate potassium salt) (PSPMK) brushes grafted onto poly(N-isopropylacrylamide)
PNIPAM-PMPC PSBMA	poly[N-isopropylacrylamide-2-methacryloyloxyethyl phosphorylcholine] (PNIPAM-PMPC)
PSBMA	poly(sulfobetaine methacrylate)
PSPMK	poly(3-sulfopropyl methacrylate potassium salt)
RA	rheumatoid arthritis
RNS	Reactive Nitrogen Species
ROS	Reactive Oxygen Species
SB-g-NBrMGs	poly(3-sulfopropyl methacrylate potassium salt) (PSPMK) brushes grafted poly(N-isopropylacrylamide)(PNIPAAm) micro-gels
SF	synovial fluid
SUV	single unilamellar vesicles
VEGF	Vascular Endothelial Growth Factor
